# Influence of Pure Aluminum and 7075 Aluminum Alloy Powder Interlayers on the Microstructural and Mechanical Properties of Diffusion-Bonded 7B04 Aluminum Alloy Joints

**DOI:** 10.3390/ma18214907

**Published:** 2025-10-27

**Authors:** Ning Wang, Chunbo Li, Lansheng Xie, Minghe Chen

**Affiliations:** 1 Engineering Technology Training Center, Nanjing University of Industry Technology, Nanjing 210023, China; 2 ZTE Corporation, Nanjing 210012, China; 19852830138@163.com; 3 Jiangsu Key Laboratory of Precision and Micro-Manufacturing Technology, Nanjing University of Aeronautics and Astronautics, Nanjing 210016, China; meelsxie@nuaa.edu.cn (L.X.); meemhchen@nuaa.edu.cn (M.C.)

**Keywords:** diffusion bonding, 7B04 aluminum alloy, powder interlayers, particle size, lap shear strength

## Abstract

Diffusion bonding (DB) of aluminum alloys faces significant technical challenges, requiring thorough surface preparation and precise control of process parameters. To enhance the joint quality of 7B04 aluminum alloy sheets, pure aluminum (Al) and 7075 aluminum alloy powders were used as interlayers. In the DB experiments, nano-sized Al powder and micro-sized 7075 powders with different particle sizes served as interlayer materials. Compared to DB without an interlayer, using powder interlayers substantially reduced the bonding temperature while improving overall joint performance, with deformation kept below 6%. The lap shear strength (LSS) of the bonded 7B04 joints was significantly higher when 45 μm and 75 μm 7075 powders were used, compared to the 5 μm 7075 powder. The joint with a 50 nm Al powder interlayer achieved a maximum LSS of up to 220 MPa and exhibited considerably higher microhardness. Additionally, the mixed Al/7075 powder interlayer effectively decreased voids at the joint interface, contributing to increased LSS.

## 1. Introduction

The solid-state diffusion bonding (DB) technique has been developed to create high-quality joints for both similar and dissimilar materials with minimal micro-deformation [1,2,3]. Compared with other welding methods, DB is particularly useful in welding applications that require precise connections and the formation of complex parts with high accuracy, such as microchannel and narrow-channel aluminum alloy water-cooled plate heat sinks, where it plays an essential and irreplaceable role. DB offers improved design flexibility with less brittle intermetallic compound formation, reduced residual stress, decreased microstructural degradation, and minimized chemical segregation [4,5,6]. This method is widely used in manufacturing lightweight structures made of titanium, copper, and steel in the aeronautic and electronic industries [7,8]. However, aluminum alloys often present welding challenges, mainly due to the persistent oxide film and significant deformation during hot pressing [9,10]. 7B04, a high-strength aluminum alloy from the 7XXX series, is commonly used in aeronautical applications such as fuselage skins and stringers because of its exceptional strength, low density, and resistance to stress corrosion [11,12,13]. Achieving a DB joint of 7B04 with high strength remains a significant challenge for further developing the DB and superplastic forming process to manufacture complex, lightweight, integral components.

Oxide films mainly composed of Al_2_O_3_, which have a high melting point, are the primary obstacle to atom diffusion, as they inevitably form on aluminum alloy surfaces exposed to the atmosphere [9]. One way to overcome this diffusion barrier is through surface treatment and a vacuum or inert gas environment [14]. Huang et al. [9] developed a new surface treatment method where the surfaces to be joined were coated with an organic solution immediately after removing surface oxides for DB and obtained promising results under optimized bonding conditions. Zuruzi et al. [15] used SiC paper and subsequent ultrasonic cleaning in acetone for 5 min to disrupt the surface oxide layer, and the ultimate tensile strength and linear bonded ratio of 6061. Shirzadi et al. [16] used emery paper grinding containing a small amount of liquid gallium to replace the oxide film with a thin metallic layer and/or less stable oxides, which resulted in bonding strength similar to the parent 6082 aluminum alloy. Wu et al. [17] applied dehydrated alcohol for air isolation after chemical etching of AA8090, and the DB was performed in a salt bath. A vacuum [1] or protective atmosphere [18] can effectively prevent oxidation of the joining surfaces, demonstrating promising outcomes in experimental and practical applications.

DB with a sound interlayer is feasible because its composition can be flexibly adjusted to meet phase and mechanical property requirements [19]. Research has been conducted to improve the DB joint quality by adding foil and powder interlayers. Aravinda et al. [10] bonded Al2024 sheets using Fe_2_O_3_ powder interlayer in an open atmosphere. Varmazyar et al. [19] used cold-rolled copper interlayers to bond pure aluminum and magnesium bulks. The joint quality was improved by increasing the strain in the copper interlayer. An Al foil interlayer enhances element distribution and microstructure, leading to increased bonding ratio and shear strength while reducing deformation [20]. Urena et al. [21] employed an aluminum-lithium foil interlayer in the DB of AA2024 alloy reinforced with SiC particles. The interlayer plastically flows, thereby reducing voids at bonding interfaces when the thickness deformation decreased by 25%. Song et al. [22] used spark plasma diffusion welding (SPDW) to bond 5A06 aluminum alloy with an Al–20Cu–5Si–2Ni interlayer and achieved a sound joint under optimized conditions. TA1 titanium and 5052 aluminum plates were diffusion-bonded with a 1060 Al interlayer. The {110}<112> rolling texture and {001}<100> cube texture on the Al side improved strength and preserved processing capabilities [23]. Pure Al was used as an interlayer to bond 7050/7A52 and 7A62/7A52 alloys. Interfacial grain boundary migration caused by dynamic recrystallization contributed more to tensile strength improvement than element diffusion [24].

Mixed powders and foils have been widely used to enhance bonding performance. Urena et al. [25] also used Al-Cu interlayers to improve contact between both Al6061 sheet surfaces and achieve high-quality joints under suitable bonding conditions. Lee et al. [26] combined solid-state DB and transient liquid phase (TLP) DB to bond Mg/Al alloys using a Zn/Sn composite interlayer at low temperatures. Huang et al. [27] employed mixed Al–Si and Al–Cu powders as interlayers to bond SiCp/6063 metal matrix composites (MMC). The mixed powders resulted in a hypoeutectic microstructure that strengthened the joint. Hosseinabadi et al. [28] used mixed hydride/alanate nano Al-Mg powders with a 50-50 M ratio as the interlayer to improve the bond strength of alumina parts at low temperatures.

Much research has been conducted on the effects of DB temperature, time, pressure, and surface treatment on joint performance [14]. However, the impacts of powder size or foil thickness on the DB process are rarely reported. The diffusion-bonded Al/Ti alloys joint with Sn–4Ag–3.5B interlayers of different thicknesses (50 and 100 μm) was compared. The thicker interlayer led to discontinuities and thick intermetallic layers at the joint interface [1]. Guo et al. [29] used a pure nickel layer of varying thickness as the interlayer in powder metallurgy superalloy FGH98. As the interlayer becomes thinner, it is easier to achieve alloying, but this also makes the microstructure transition in the joint less uniform. Hu et al. [30] used Ti foil thickness (5 µm, 10 µm, 20 µm) as an interlayer, which had a relatively minor influence on the SiC ceramics DB. Thinner Ti interlayers (5 µm and 10 µm) produced Si-rich phases in the central region, resulting in stress concentration and a reduction in the strength of the joints. Thin films (1 µm), commercial titanium foils (5 µm), and no interlayer were used in joining Ti6Al4V to Alumina by DB. The Ti thin film improved the shear strength of the joints under optimal bonding parameters [31]. By spark plasma sintering (SPS), the DB experiments of diamond/Cu composites with different thickness Cr interlayers were performed. It shows that the shear strength increases and then decreases with increasing interlayer thickness [32].

In this study, pure Al and 7075 powders of various sizes, along with mixed powders at different weight ratios, are used as interlayers in vacuum diffusion bonding of 7B04 sheets. The lap shear strength (LSS), microhardness, and microstructures are measured to evaluate the quality of diffusion-bonded 7B04 joints with different powder interlayers.

## 2. Materials and Methods

### 2.1. Materials

1.2 mm thick 7B04-T74 aluminum alloy sheets were used as the base metal. The shear strength and tensile strength of 7B04 are 260 MPa and 424 MPa, respectively. Pure Al powders (50 nm, 1 µm), 7075 powders (5 µm, 45 µm, 75 µm), and mixed Al (1 µm)/7075 (75 µm) powders of different weight ratios were used as interlayers for 7B04 aluminum alloy DB. Both pure Al and 7075 alloy exhibit excellent chemical compatibility with the 7B04 base metal, reducing the risk of forming brittle intermetallic compounds at the interface. As shown in Table 1, the chemical composition of 7075 alloy closely resembles 7B04 (both in the Al-Zn-Mg-Cu series), ensuring microstructural and mechanical uniformity in the joint. A selection of particle sizes (50 nm, 1 μm, 5 μm, 45 μm, 75 μm) was made to systematically examine how particle size affects joint properties, based on previous studies [18,29,30,31,32]. Fine powders (nano/micro) promote diffusion but may tend to agglomerate, while coarser powders improve flowability and decrease agglomeration but might require greater deformation to ensure full contact. This choice is supported by prior research using similar interlayers for aluminum alloy diffusion bonding. Nano-sized pure Al powder provides a highly pure diffusion pathway due to its high atomic mobility and lack of alloying elements that could hinder diffusion. The nanoscale particles, with their large surface area-to-volume ratio, greatly enhance atomic diffusion rates and facilitate void closure at the bonding interface.

### 2.2. Experimental Method

The 7B04 aluminum alloy was polished with 1500 # sandpaper, then immersed in a 5% NaOH solution for 5 min and a 30% HNO_3_ solution for 3 min, followed by ultrasonic cleaning in anhydrous ethanol. Finally, a 15 min plasma surface cleaning was performed using a plasma surface treatment machine TS-PL05 (Shenzhen Tonson Tech Automation Equipment Co., LTD., Shenzhen, China) with a 40 KHz power supply, 600 W radio frequency power, and a 400 mL/min argon gas flow rate to remove oxide and improve surface roughness. The average Ra value increased from 0.409 ± 0.024 µm to 0.436 ± 0.032 µm after plasma treatment.

The DB process was carried out in a press with a vacuum furnace at a temperature of 515 °C under a pressure of 4.4 MPa, held for 7.5 h. The experimental condition was optimized based on previous research. The parameters were derived from previous 7B04 DB process optimization work aimed at achieving high LSS. A base pressure of ≤5.0 × 10^−4^ Pa was achieved and maintained before heating started. During the vacuum process, the mechanical pump is first turned on for initial exhaust. After about 15 min, the pressure inside the vacuum chamber drops to roughly 10 Pa. Once the pressure falls below 10 Pa, the molecular pump is activated to begin operation. Total pump-down time to reach base pressure is 45–60 min. The pressure remains below 5.0 × 10^−3^ Pa during heating.

The powders used as interlayers in the experiments are shown in Table 2. Diethylene glycol dibutyl ether was used to protect the aluminum from oxidation. The diethylene glycol dibutyl ether was pre-sucked into a syringe and injected into the vacuum-packed aluminum alloy micron powder without opening the package, with a mixing ratio of 0.04 mL/mg. The powder mixed with the protective agent was subjected to ultrasonic treatment for 5 min and then uniformly coated on the 7B04 base metal via a syringe at 0.15 mL/cm^2^. Diethylene glycol dibutyl ether is a colorless liquid with a boiling point of 256 °C. It does not vaporize at room temperature and has good adsorption properties. During diffusion bonding, when the welding temperature increases, the protective agent heats up to its boiling point, vaporizes, and escapes from the vacuum furnace.

**Figure 1:**
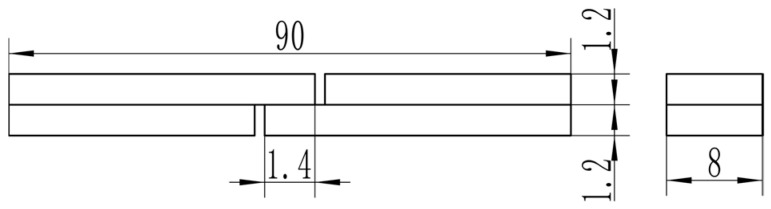
Dimensions of LSS test specimens.

The LSS test specimen is shown in Figure 1. A UTM 5504X electronic universal testing machine (SUNSTEST Inc., Shenzhen, China) was used for the non-standard LSS test. The test speed is 1 mm/min. No anti-bending fixture was used because the lap length is only 1.4 mm, which was determined through prior experiments to guarantee fracture in the overlap area. For each experimental condition, a minimum of three valid replicates were tested. After tensile testing, the LSS specimens were ultrasonically cleaned in an acetone solution. The fracture surface was then examined using a Regulus 8220 (Hitachi High-Tech Corporation, Tokyo, Japan) scanning electron microscope (SEM). Meanwhile, the interface of the joint was scanned by point and line using an energy dispersive spectrometer (EDS) to analyze element diffusion at the interface between the base material and the intermediate layer.

Microhardness of the diffusion-bonded 7B04 joints was measured using an HVS-1000A (Laizhou MetalReader Test Instrument Co., Ltd., Laizhou, China) with a 0.98 N force applied for 10 s. The metallographic samples cut with an electric discharge wire were polished with sandpapers (#400–#2500) and diamond grinding pastes (W3.5, W2.5, and W0.5), then immersed in an etching solution (1 mL HF, 1.5 mL HCl, 2.5 mL HNO_3_, 95 mL H_2_O) for 10 s. The microstructure was observed with a metallographic microscope MR5000 (Nanjing Ruiyuan Optical Instrument Co., Ltd., Nanjing, China).

## 3. Results and Discussions

### 3.1. Influence of Powder Size on DB Joints

#### 3.1.1. Microstructure of the Joint

**Figure 2:**
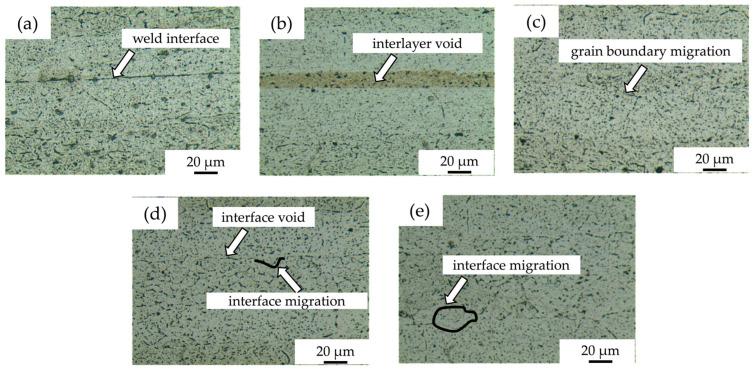
DB interface microstructure of 7B04 with different powder interlayers: (**a**) no interlayer; (**b**) 50 nm Al; (**c**) 5 μm 7075; (**d**) 45 μm 7075; (**e**) 75 μm 7075.

Figure 2a shows the microstructure of diffusion-bonded joints without powder interlayers, with obvious weld defects and a lower bond rate compared to the joints with powder interlayers. Figure 2b shows a joint obtained with a 50 nm Al powder layer, which appears fine with no powder grain boundaries and a high weld rate. The diffusion interface between aluminum powder and substrates forms effective metallurgical bonds, with minimal voids, and a higher Al concentration in the nano-aluminum layer facilitates atom diffusion, forming a denser interface. However, residual pores exist due to incomplete mixing and aggregation from viscosity effects of the residual antioxidant. Figure 2c–e display the microstructures of joint diffusion bonded with intermediate layers of 5 μm, 45 μm, and 75 μm 7075 alloy powders, respectively. The 5 μm 7075 powder interlayer shows no obvious interface voids, fewer and finer poles in the powder interlayer than the 50 nm Al. The dotted lines in Figure 2d,e indicate the fusion between the matrix grains and the powder interlayer, increasing the bonding interface quality. The grain boundaries are more obvious for the 45 μm and 75 μm 7075 alloy powder interlayers due to a larger grain size and fewer grain boundaries. The intermediate layer powder deforms and flows under pressure. Since the applied force is unidirectional and axial, it cannot guarantee full contact between the powders. Fine powder in the middle layer tends to agglomerate, creating isolated, closed voids. For larger powders, narrow, fine, curved holes can be observed along the grain boundaries.

Bonding 7B04 using 7075 powder involves three main stages. First, powders slide relative to each other and the surface under pressure. The gaps are filled, increasing density, which improves contact and diffusion pathways. Second, diffusion occurs between the powder interlayers and base metal, with elements diffusing under heat and pressure, strengthening void closure and causing grain boundaries to dissolve. Third, grain growth causes boundaries to migrate with extended bonding, resulting in uneven interfaces, reduced strength, slight displacement, and some holes between powders and the base material.

#### 3.1.2. Element Diffusion at the Joint

**Figure 3:**
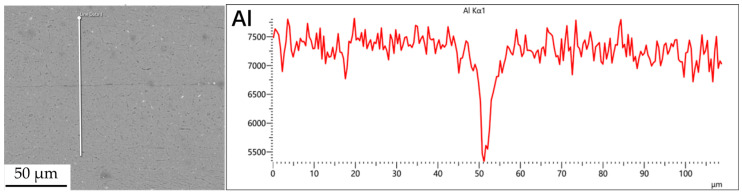
Scan of element distribution at diffusion joints of 7B04 with no powder interlayer.

**Figure 4:**
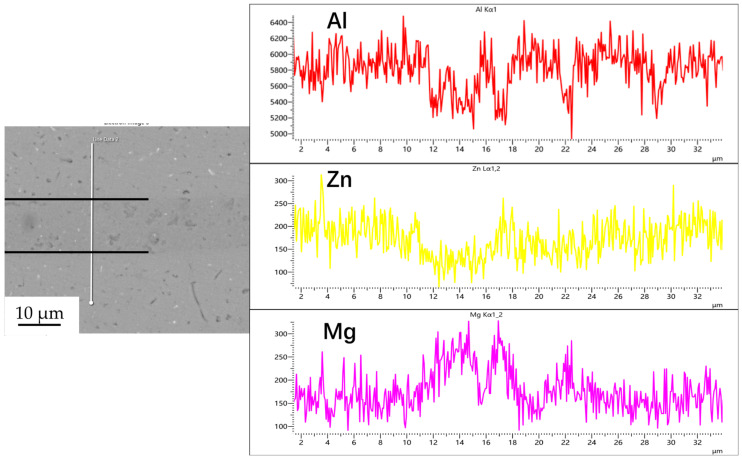
Line scan of element distribution at diffusion joints of 7B04 with 50 nm Al powder interlayer.

**Figure 5:**
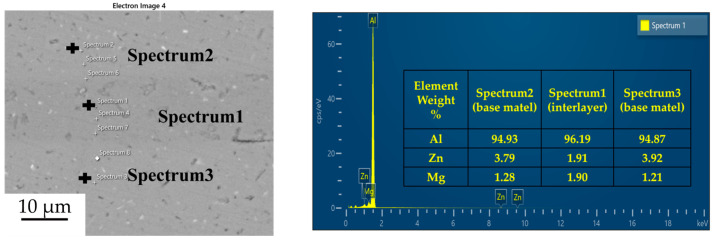
Spot scan of element distribution at diffusion joints of 7B04 with 50 nm Al powder interlayer.

**Figure 6:**
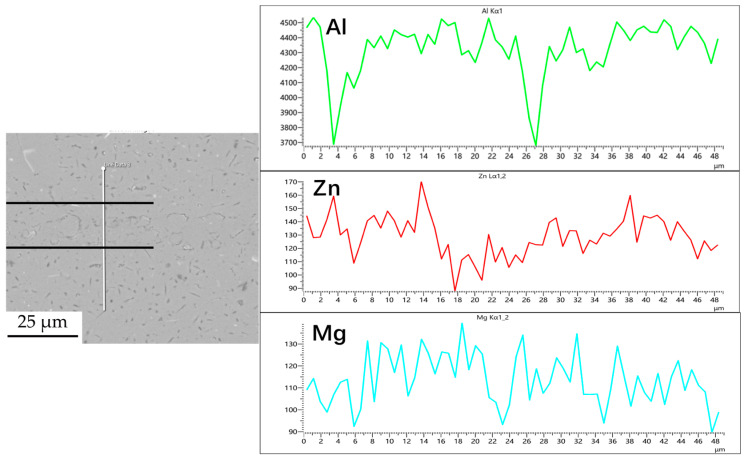
Line scan of element distribution at diffusion joints of 7B04 with 5 μm 7075 powder interlayer.

Figure 3, Figure 4, Figure 5 and Figure 6 illustrate the distribution of elements across the interface perpendicular to the diffusion joints 7B04 with various powder interlayers. The composition of the base material 7B04 and the intermediate layer 7075 powder was similar, with no formation of special phases or compounds. Figure 3 shows the element distributions at the joint without powder interlayers. It is clear that there is no difference in the distribution of Al elements on both sides of the base material. Al elements are detected in the middle weld area, but their concentration is significantly lower than that in the base materials on either side. This difference in Al concentration indicates mutual diffusion of elements at the interface and limited bonding. Additionally, Zn, Mg, and Cu elements are detected in the middle region. Figure 4 depicts the element distribution at the diffusion joint with a 50 nm Al powder interlayer. There is no significant change in Al content from one side of the base material to the powder interlayer and then to the other side. The concentration differences during the diffusion bonding process facilitate the diffusion of Al. Simultaneously, small amounts of Zn, Mg, and Cu diffuse into the powder interlayer. Variations in element concentration promote mutual atomic diffusion at the interface, increase the diffusion bonding flux, and enhance metallurgical bonding between the powder interlayer and the base material. From Figure 5, it is evident that the Al content in the powder interlayer is slightly higher than in the base material. Mg and Zn are present in the middle layer, with Mg diffusing into the middle layer more extensively than Zn, due to its higher diffusion coefficient in Al. Figure 6 shows the element distribution of the joint with a 50 nm 7075 powder interlayer. The Al content remains relatively uniform along the line scan; the two areas with reduced Al might be sites of material pit defects. There is no clear pattern or characteristic of elemental diffusion, and the alloying elements are gradually homogenized throughout the entire joint interface. Similar element distribution results were obtained for 7B04 joints with 45 μm and 75 μm 7075 powder interlayers. Additionally, it is notable that the oxygen content at the diffusion interface is very low, indicating that the base metal and powder are well protected during diffusion bonding oxidation.

#### 3.1.3. Microhardness

**Figure 7:**
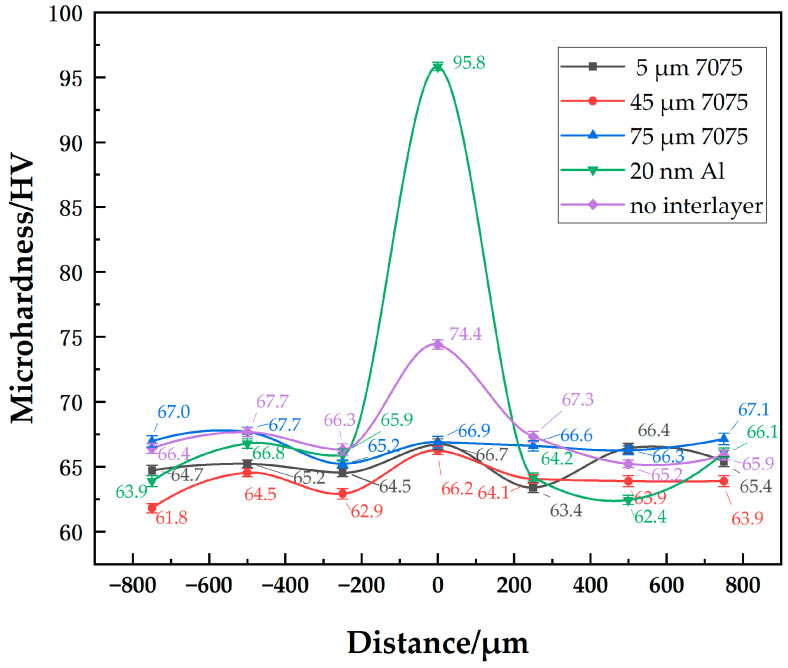
Distribution of microhardness at joint of 7B04 with different powder interlayers.

The microhardness distributions of joints with different-sized powder interlayers are shown in Figure 7. They show little difference in the hardness distribution of joints with 5 μm, 45 μm, and 75 μm 7075 powder interlayers. Since the properties of 7B04 and 7075 are similar, the hardness of the powder interlayer at well diffusion-bonded joints is comparable to that of the base materials. At a process set of 515 °C—7.5 h—4.4 MPa, the hardness of the 7B04 base material and powder interlayer is around 65 HV. The hardness of 73 HV in the middle of the diffusion joint without an powder interlayer is higher because surface micro-deformation occurs in the directly contacted 7B04 base material when it is compressed, and the weld seam is narrow. The indenter’s action area mainly covers the middle diffusion layer, resulting in higher hardness due to the hardening effect. The hardness of the 50 nm Al powder interlayer reaches 96 HV. The significantly higher microhardness of the joint with the 50 nm Al powder interlayer, compared to the base metal, is mainly due to the Hall-Petch strengthening mechanism. The nano-sized powder particles create a very fine-grained structure within the powder interlayer after bonding. According to the Hall-Petch relationship, a decrease in grain size leads to an increase in yield strength and hardness, as grain boundaries act as barriers to dislocation motion. Although some residual pores exist, the high density of grain boundaries in this nano-structured powder interlayer results in a substantial hardening effect. The microhardness values of base metal with different powder interlayers range from 61.8 to 67.7. Material inhomogeneity and systematic measurement errors are fundamental factors contributing to the differences in the base metal microhardness.

#### 3.1.4. LSS and Fracture Analysis

**Figure 8:**
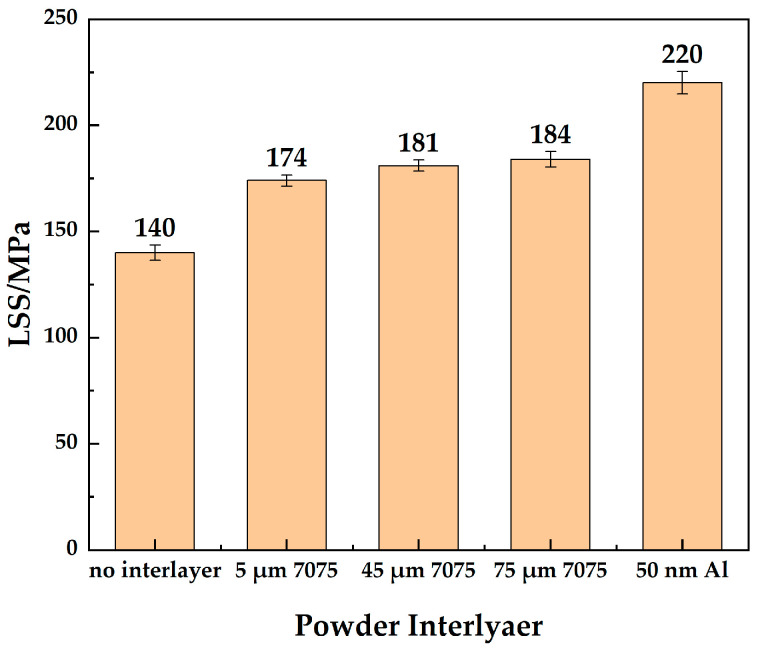
LSS of 7B04 joints with different powder interlayers.

As shown in Figure 8, the LSS of the joint without an powder interlayer was 140 MPa. The shear strength of the joint with the 5 μm 7075 powder interlayer was 174 MPa, slightly lower than that of the 45 μm and 75 μm powder interlayers, which were 181 MPa and 184 MPa, respectively. The joint with the 50 nm Al powder interlayer has the highest LSS, reaching 220 MPa, due to its higher density. The thickness difference before and after bonding was measured. The thickness of the LSS sample was measured by micrometer with an accuracy of ±1 µm before and after DB (t_1_ and t_2_). The deformation rate was calculated (t_1_ − t_2_)/t_1_ × 100%. The deformation rates along the thickness direction were less than 6%, as shown in Table 3. The overall deformation of the bonding sample was small and met the requirements.

**Figure 9:**
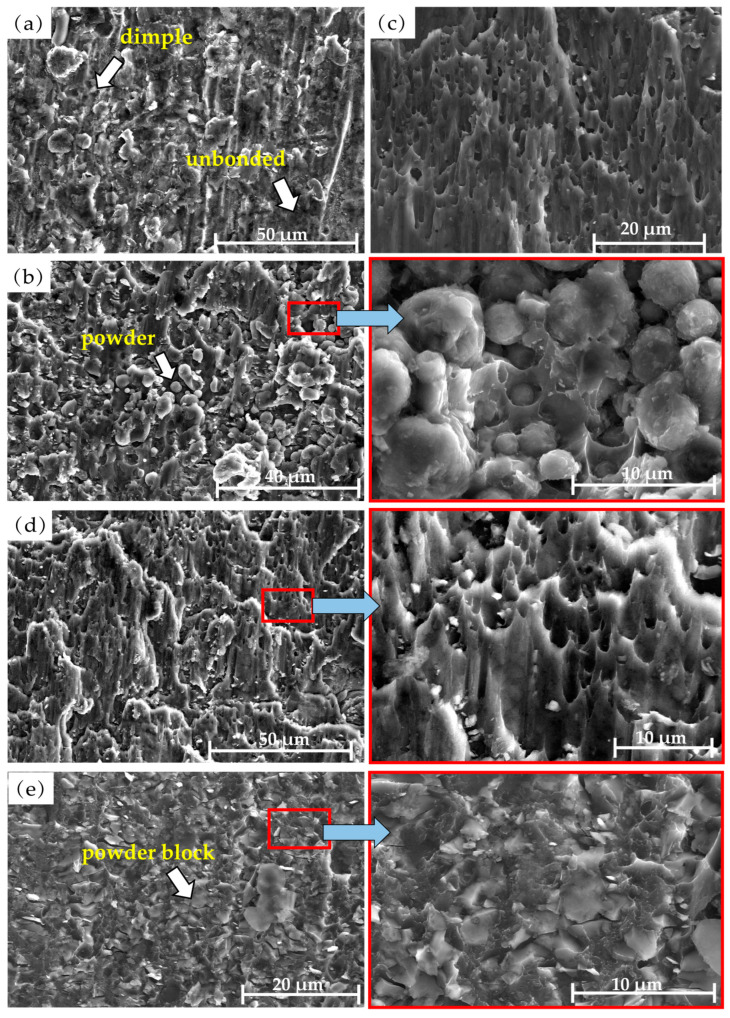
LSS fracture morphology of 7B04 joints with different powder interlayers: (**a**) no interlayer; (**b**) 5 μm 7075; (**c**) 45 μm 7075; (**d**) 75 μm 7075; (**e**) 50 nm Al.

Figure 9 shows the SEM fracture morphology of the LSS test samples of the diffusion-bonded 7B04. Figure 9a displays the fracture morphology without an powder interlayer. The darker flat area indicates the unwelded region. It exhibits a transgranular dissociation fracture with dissociation steps of varying heights, and the fracture type is characteristic of brittle fracture. Additionally, a few dimples can be seen at the fracture site. Therefore, the fracture of the 7B04 aluminum alloy diffusion-bonded specimens without an powder interlayer was a mixed fracture mode primarily driven by dissociative brittle fracture.

Figure 9b shows the fracture morphology of the shear specimen with a 5 μm 7075 powder interlayer. Fracture failure occurred at the interface between the powder interlayer and the base material, displaying a mixture of dimples and dissociations. The powder bonded to the matrix was separated from the base material under shear stress, forming equiaxed dimples. Many powders have not separated from the base material, indicating higher bonding strength. Some powder particles have equiaxed dimples facing different directions. The magnified image shows that the dimples vary in size and depth. The formation mechanism of the dimples is mainly void aggregation fracture in the unbonded locations. Although the powder interlayer of 7075 in metallographic Figure 2c has achieved metallurgical bonding, powder agglomeration causes the overall density to be low, leaving many voids between the powders. When the shear specimen is subjected to shear stress, these unclosed voids will expand, and the cross-sectional area between adjacent voids will shrink, connect, and merge to form new voids, eventually leading to a dimple that results in ductile fracture.

Figure 9c,d display the fracture surface features of the shear specimens with 45 μm and 75 μm 7075 powder interlayers. The specimens did not fracture at the intermediate layer but at the interface between the powder interlayer and the base metal. This occurs because, compared to fine-grained powder, larger powder particles are less prone to physical agglomeration and lack obvious isolated closed voids. However, narrow voids can form along the grain boundaries between some powders due to insufficient contact. Additionally, coarse-grained powder tends to have voids at the interface between the intermediate layer and the matrix, which are weaker and lead to fracture at the interface. Under shear stress, the powder attached to the diffusion layer begins to detach from the matrix, and the voids are progressively elongated, while the powder with stronger bonding to the matrix remains attached. The fracture profiles of the 45 μm and 75 μm powder interlayers are similar, characterized by parabolic shear dimples. The abundance and density of shear dimples, along with the darker color at the bottom indicating greater dimple depth, reflect a typical ductile fracture.

Figure 9e shows the shear fracture morphology of the specimen with a 50 nm Al powder interlayer. The base metal is densely covered with nano powder blocks of different sizes. These lumps are embedded in the base material after being subjected to shear stress, presenting an irregular curved surface. The voids in the powder interlayer lead to relatively low strength. Fracture occurs preferably in these areas, indicating that a higher metallurgical bond is formed between the nano-aluminum powder and the base material. The areas where the nano-aluminum powder separates from the base material show equiaxed dimples.

In summary, the powder interlayer altered the fracture failure mode of the 7B04 joint under shear load, increasing the LSS. The fine-grained aluminum alloy powder separates from the base material and forms equiaxed dimples on it. Some powder remains attached to the base, displaying equiaxed dimples with different orientations. The nano Al powder interlayer bonds well with the base material. The base is embedded with nano-powders of various sizes, showing irregular curved surfaces after the LSS test. Coarse-diameter powder is sheared off, creating elongated, deep, and dense parabolic dimples.

### 3.2. DB with the Mixed Al/7075 Powder Interlayer

#### 3.2.1. Microstructure of the Joint

**Figure 10:**
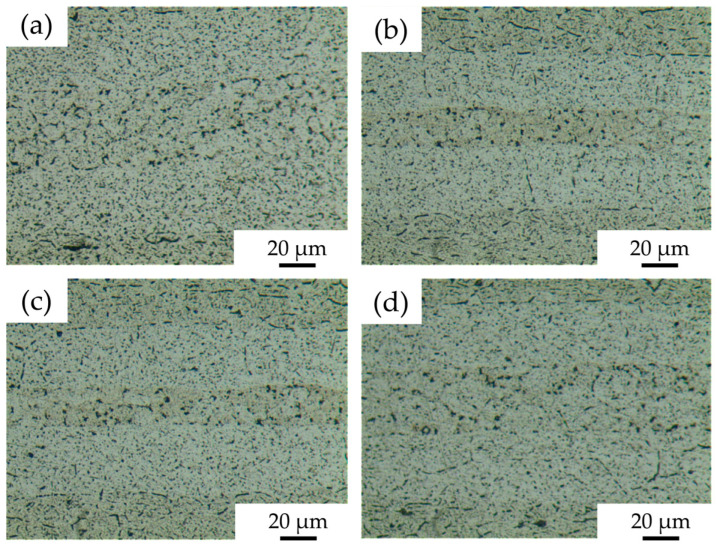
DB interface microstructure of 7B04 with mixed Al/7075 powder interlayers of different weight ratios: (**a**) 25%; (**b**) 50%; (**c**) 75%; (**d**) 100%.

Microstructures of 7B04 diffusion joints with different Al/7075 powder mix ratios obtained at process parameters of 515 °C—7.5 h—4.4 MPa are shown in Figure 10. In Figure 10a, with a mixing ratio of 25%, no obvious Al powder was observed at the diffusion interface. The grain boundaries between the 7075 aluminum alloy powders are weakened, and small voids remain in the powder interlayer. Compared to the microstructure of the joint without Al powder, the interface voids are reduced. In Figure 10b, with a 50% mix ratio, 7075 powder is surrounded by Al powder with no obvious grain boundaries. The interface voids between the powder interlayer and the base material are significantly decreased. The Al powder is mainly aggregated due to uneven distribution during manual coating, resulting in some tiny voids and agglomeration. The metallographic microscopic characteristics of the joints at 75% and 50% mix ratios are similar. In Figure 10d, at a 100% mix ratio, neither Al nor 7075 powder is particularly prominent. The entire diffusion surface is more uniform, with small voids still present but fewer in number.

#### 3.2.2. Microhardness

**Figure 11:**
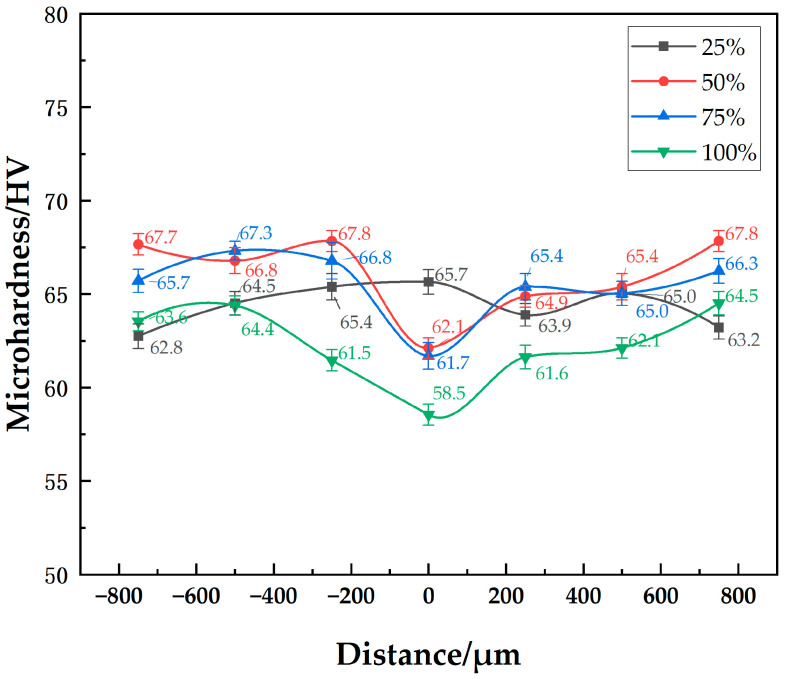
Distribution of microhardness at joint of 7B04 with mixed Al/7075 powder interlayers of different weight ratios.

The microhardness distribution of the 7B04 joints with mixed Al/7075 powder interlayers of different mass ratios is shown in Figure 11. As the Al/7075 powder mixture ratio increases, the powder interlayer’s hardness decreases. The lowest microhardness was 58 HV at a 100% mixed ratio, showing no significant decrease compared to the powder interlayer microhardness of 7075 powder at around 65 HV. The decrease in microhardness observed with the mixed Al/7075 powder interlayer, especially as the proportion of soft, micro-sized Al powder increases, is due to a different mechanism. The 7075 alloy powder itself has a hardness similar to the 7B04 base metal. However, the incorporated pure Al powder (1 µm) is significantly softer. As the mixing ratio of Al powder increases, the overall hardness of the powder interlayer composite decreases because the softer phase (Al) governs the mechanical response, leading to a lower average microhardness value. The microhardness values of base metal with different mixed Al/7075 powder interlayers range from 61.5 to 67.8. Material inhomogeneity and systematic measurement errors are fundamental factors contributing to the differences in the base metal microhardness.

#### 3.2.3. LSS and Fracture Analysis

**Figure 12:**
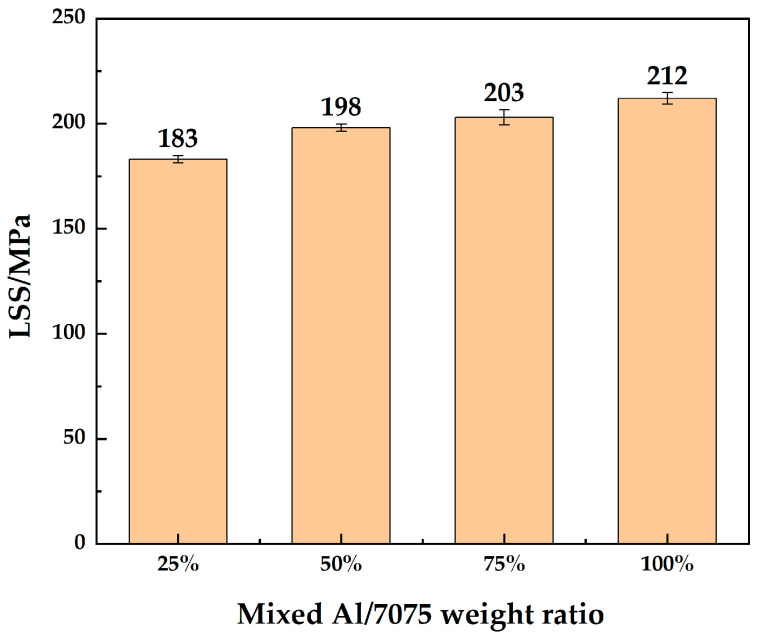
LSS of 7B04 joints with mixed Al/7075 powder interlayers of different weight ratios.

LSS test results of 7B04 DB joints with mixed Al/7075 powder interlayers of different mixing ratios are shown in Figure 12. The LSS improved with the addition of Al powder. The Al/7075 powders with a mixing ratio of 25% make little difference in LSS compared to 7075 powder. The LSS of joints with 50% and 75% mixed Al/7075 powder interlayers are similar. The LSS of the joint reached 212 MPa at a 100% mixing ratio. Deformation rates of the joints are listed in Table 4. The diffusion-bonded 7B04 samples, obtained by adding mixed Al/7075 powder interlayers, exhibit minimal deformation, and the dimensional accuracy was controlled to meet test requirements.

**Figure 13:**
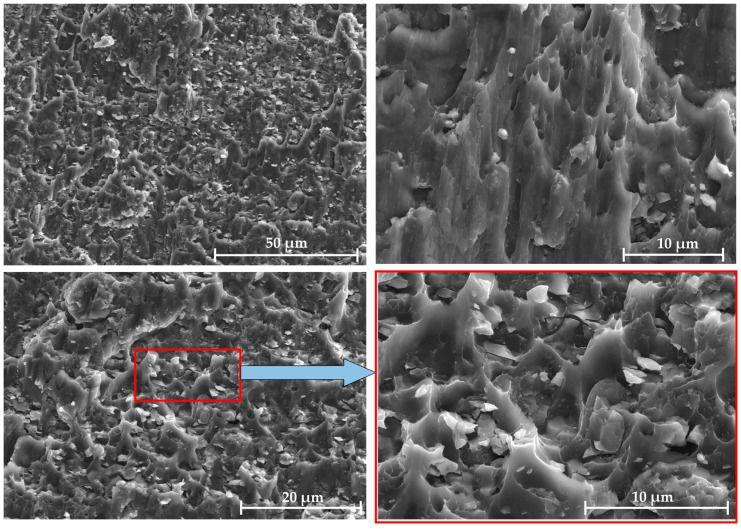
Fracture morphology of 7B04 joint with mixed Al/7075 powder at 100% ratio.

Similar fracture surfaces of the LSS test specimens with Al/7075 powders at different mixing ratios were observed. Taking the 100% mixing ratio sample as an example, as shown in Figure 13, the fracture appearance of the Al/7075 mixed powder displays polymorphism. The Al and 7075 powder particles in the powder interlayer both bond to the base material. The 45 μm 7075 aluminum alloy powder is separated from the base material under shear stress, forming elongated parabolic dimples. Micro-aluminum powder has a weaker bond with the base material compared to nano-aluminum powder and does not embed in the base material. Random dimples of different sizes are formed when the micro-aluminum powder separates from the base material.

### 3.3. DB Mechanism of 7B04 with Powder Interlayer

**Figure 14:**
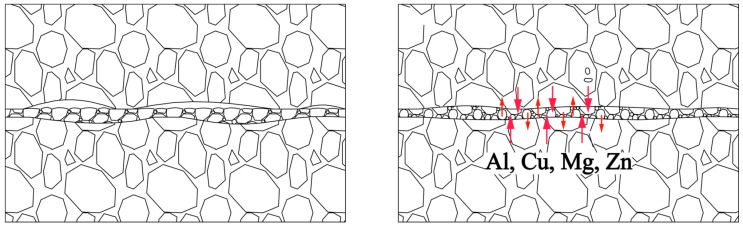
Model of DB interface with powder interlayer.

Adding the powder interlayer changes the physical contact between the base metal surface during DB, allowing diffusion bonding with minimal deformation and high shear strength. The rough surface of the base material can produce sufficient plastic deformation when pressure is applied, and the associated creep helps break the oxide film. However, a deeper groove makes close contact between the base materials more difficult. The limited effective physical contact area affects the quality of the diffusion bonding at the joint. The addition of the powder alters the contact with the base metal, as illustrated by the 7B04 DB interface model shown in Figure 14. Under pressure, the powder undergoes plastic deformation as it flows to fill the gaps between the base materials, ensuring full contact with the base material.

**Figure 15:**
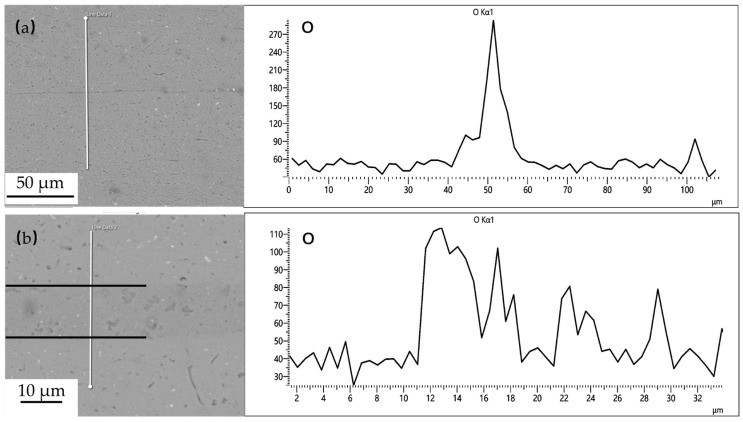
O distribution at the 7B04 joints with: (**a**) no powder interlayer; (**b**) 50 nm Al powder interlayer.

Powder interlayers are beneficial for breaking the oxide film on the aluminum alloy surface during DB. After pre-weld surface treatment, the oxide film on the aluminum alloy surface during the test is relatively thin. The aluminum alloy powder has higher hardness, which improves the breaking of the oxide film under pressure, facilitating the formation of atomic diffusion channels. The grains of the powder interlayer powder in contact with the base material grow toward the base material and undergo grain boundary migration, effectively preventing the re-formation of the oxide film. As shown in Figure 15, the distribution of oxygen at the interface of the joint with no powder interlayer and the 50 nm Al powder interlayer was tested by EDS. The oxygen concentration at the non- powder interlayer bonding interface is significantly higher than that at the 50 nm Al powder interlayer, indicating that the degree of oxidation in DB with the non-powder interlayer interface is greater than with the powder interlayer.

The pre-bonding surface treatment is essential and effective for removing the initial, thick, stable oxide film. The O element detected at the interface in Figure 15 indicates that minimal re-oxidation occurs during the heating cycle before intimate contact is established, and from very thin native oxides that re-form instantly on ultra-clean surfaces with minimal exposure. The significantly lower oxygen concentration at the interface with the 50 nm Al powder interlayer, compared to the no powder interlayer joint, demonstrates the additional protective benefit of the powder interlayer. The powder particles can physically disrupt and disperse any emerging oxide layers during deformation under pressure, creating more direct metal-to-metal contact pathways than in the direct base-metal-to-base-metal contact scenario.

The main advantage of nano-powder is its exceptionally high specific surface area and associated high surface energy. This results in a significantly greater number of atomic diffusion pathways compared to micro-sized powders. Enhanced Surface Diffusion: Atoms on the surface of nano-powders have a much higher energy state and mobility, leading to faster and more complete consolidation of the powder interlayer. This forms a more continuous and denser diffusion interface. The small radius of nano-particles creates a high chemical potential gradient, providing a stronger driving force for atomic diffusion, which facilitates pore closure and metallurgical bonding. Finer particles can deform and flow more effectively to fill the micro-asperities on the prepared base metal surfaces, ensuring a more intimate and continuous contact area. This maximizes the effective bonding surface, which is directly related to the joint strength. In contrast, larger, stiffer micro-sized powders cannot conform as well to the surface topography. This can lead to incomplete contact at certain points, creating isolated or curved voids. The 50 nm powder used was high-purity aluminum. When placed between the 7B04 alloy substrates, it establishes a steep aluminum concentration gradient across the interface. It serves as a strong driver for Al atoms to diffuse from the powder interlayer into the base metal and vice versa, significantly increasing the diffusion rate. While the 7075 powder has a composition similar to 7B04, the concentration gradients for individual alloying elements are much smaller, resulting in a slower and less intense interdiffusion process.

The mixed powder interlayer creates a bimodal particle size distribution. The smaller Al powder particles serve as fillers, effectively filling the gaps between the larger 7075 powder particles. This optimized packing significantly reduces the initial void volume within the powder interlayer before and during the application of pressure. The introduction of pure Al powder also creates a high concentration gradient for aluminum atoms across the interface between the pure Al particles and the surrounding 7075 particles or the 7B04 base metal, which increases the diffusion flux of aluminum atoms. This enhanced atomic mobility accelerates the bonding process at the interfaces between powder particles and between the powder interlayer and the base metal. The mixed powder system works by combining mechanical filling with enhanced chemical diffusion. This dual action ensures better initial contact and promotes a more complete metallurgical bonding process, effectively reducing the number and size of voids at the joint interface.

## 4. Conclusions

DB experiments on 7B04 aluminum alloy with interlayers made of powders of various sizes and mixing ratios are conducted. The performance of the diffusion joint was evaluated from microstructure analysis, interface element diffusion, microhardness distribution, LSS, and deformation rate. The conclusions are as follows:

1. The introduction of powder interlayers considerably improves joint quality compared to direct diffusion bonding without an powder interlayer. The joint made with a 50 nm pure aluminum powder interlayer showed superior performance, reaching a maximum LSS of 220 MPa and a microhardness of 96 HV. It is concluded that nano-sized aluminum powder is the most effective powder interlayer material under the tested bonding conditions (515 °C, 7.5 h, 4.4 MPa), providing optimal interface bonding and mechanical properties.
2. Microstructural analysis showed that joints bonded with micro-sized 7075 aluminum alloy powders (45 μm and 75 μm) also demonstrated high quality, with LSS values of 181 MPa and 184 MPa, respectively. These joints exhibited improved interfacial continuity and fewer defects compared to the joint with 5 μm 7075 powder. Therefore, it is concluded that within the micro-scale range, larger particle sizes (45–75 μm) of 7075 alloy powder are more effective for creating strong diffusion-bonded joints than finer powders (5 μm), which tend to agglomerate and form voids.
3. The use of mixed Al/7075 powder interlayers changed the joint microstructure and performance. As the amount of aluminum powder in the mixture increased, the grain boundaries within the powder interlayer became less clear, and the LSS improved accordingly. The joint with a 100% Al powder (1 μm) interlayer reached an LSS of 212 MPa. This indicates that adding aluminum powder enhances interfacial bonding and strength enhancement.
4. Elemental distribution analysis confirmed mutual diffusion of alloying elements (Zn, Mg, Cu) across the bonding interface for all powder interlayer types. The concentration gradients facilitated atomic interdiffusion, contributing to metallurgical bonding. The measured deformation rates for all bonded specimens remained below 6%. It is concluded that the powder interlayer strategy effectively promotes elemental interdiffusion while successfully controlling deformation, making it suitable for applications requiring dimensional accuracy.

## Figures and Tables

**Table 1:** Chemical composition (wt%) of 7B04 and 7075 aluminum alloys [33,34].

Element	Zn	Mg	Cu	Cr	Fe	Mn	Si	Al
7B04	5.0–6.5	1.8–2.8	1.4–2.0	0.1–0.25	0.05–0.25	0.2–0.6	0.1	Bal
7075	5.1–6.1	2.1–2.9	1.2–2.0	0.16–0.28	0.5	0.3	0.4	Bal

**Table 2:** Powders used as interlayers in the 7B04 DB.

Powder	Particle Size/Weight Ratio
No Powder	-
Pure Al	50 nm
7075	5 µm, 45 µm, 75 µm
Mixed Al (1 µm)/7075 (75 µm)	25%, 50%, 75%, 100%

**Table 3:** Deformation rates of bonded specimens with different powder interlayers.

Powder	No Powder	5 μm 7075	45 μm 7075	75 μm 7075	50 nm Al
t_1_ − 1 (mm)	2.574	2.562	2.556	2.591	2.571
t_1_ − 2 (mm)	2.575	2.583	2.583	2.582	2.553
t_1_ − 3 (mm)	2.588	2.557	2.574	2.588	2.561
t_2_ − 1 (mm)	2.429	2.430	2.441	2.500	2.441
t_2_ − 2 (mm)	2.427	2.447	2.467	2.496	2.427
t_2_ − 3 (mm)	2.440	2.422	2.460	2.499	2.436
Average Deformation rate (%)	5.70	5.23	4.47	3.43	4.96
standard deviation (%)	0.06	0.08	0.05	0.10	0.09

**Table 4:** Deformation rates of bonded specimens with mixed Al/7075 powder interlayers of different weight ratios.

Al/7075 Weight Ratio (%)	25	50	75	100
t_1_ − 1 (mm)	2.592	2.578	2.556	2.564
t_1_ − 2 (mm)	2.602	2.574	2.59	2.567
t_1_ − 3 (mm)	2.598	2.574	2.592	2.582
t_2_ − 1 (mm)	2.503	2.512	2.529	2.517
t_2_ − 2 (mm)	2.505	2.508	2.557	2.515
t_2_ − 3 (mm)	2.503	2.505	2.558	2.523
Average Deformation rate (%)	3.60	2.60	1.23	2.05
standard deviation (%)	0.14	0.07	0.14	0.22

## Data Availability

The original contributions presented in this study are included in the article. Further inquiries can be directed to the corresponding author.

## Abbreviations

The following abbreviations are used in this manuscript:

DB	Diffusion bonding
Al	Aluminum
LSS	Lap shear strength
SPDW	Spark plasma diffusion welding
TLP	Transient liquid phase
MMC	Metal matrix composites
SPS	Spark plasma sintering
SEM	Scanning electron microscope
EDS	Energy dispersive spectrometer
